# Study on Quench Sensitivity during Isothermal Treatment of 7A65 Aluminum Alloy

**DOI:** 10.3390/ma17010193

**Published:** 2023-12-29

**Authors:** Chen Li, Liangliang Bao, Ke Huang, Shiquan Huang

**Affiliations:** 1 Research Institute of Light Alloy, Central South University, Changsha 410083, China; lichen_1983_2002@163.com (C.L.); liangbao1995@163.com (L.B.); 2 AVIC Xi’an Aircraft Industry Group Company Ltd., Xi’an 710089, China; 3 State Key Laboratory of Precision Manufacturing for Extreme Service Performance, Changsha 410083, China

**Keywords:** 7A65 aluminum alloy, quenching sensitivity, TTT curve, TTP curve, premature precipitation

## Abstract

The quenching sensitivity of 7A65 aluminum alloy was investigated using interrupted quenching experiments. The time–temperature transformation (TTT) and time–temperature performance (TTP) curves of the alloy were determined. The results indicate that the nose temperature is about 320 °C and the quenching sensitivity temperature range is from 240 °C to 360 °C. During the isothermal treatment, the supersaturated solid solution resolves to the equilibrium phase of η (MgZn_2_), and the precipitation rate is the largest at about 320 °C. Through transmission electron microscope (TEM) observation and X-ray diffraction (XRD) tests, it was found that with the extension of the isothermal holding time, the originally dispersed η’ phase gradually decreases until disappear, and the number of η phase increases and gradually grows up at the grain boundary or around the Al3Zr particles. The rod-like η phase at the grain boundary is distributed from discontinuous distribution to chain-like continuous distribution, and the precipitation free zone (PFZ) is gradually generated and widened as the holding time is extended. At the nose temperature, the driving force of nucleation is high, and the diffusion rate is fast, which promotes the precipitation and growth of η phases. The coarse η phase weakens the mechanical properties. According to the results, it is recommended to increase the cooling rate at the sensitivity temperature range to reduce the precipitation of the η phase and decrease the quenching cooling rate from solution temperature to 360 °C to reduce residual stresses in the components.

## 1. Introduction

Al-Zn-Mg-Cu aluminum alloys are widely utilized structural materials in the aviation industry owing to their notable attributes, including high specific strength, hardness, corrosion resistance, toughness, and superior processing performance [1,2,3]. As a heat-treatable alloy, the desired mechanical properties can be achieved through solution hardening followed by aging treatment [4,5]. Typically, higher cooling is employed to obtain an oversaturated solid solution for the subsequent aging treatment, but results in high residual stresses being introduced [6,7,8]. Nonetheless, reducing the quench cooling rate can lead to severe premature precipitation, resulting in a degradation in the mechanical properties of the component [9]. Hence, achieving an equilibrium between mechanical properties and residual stresses is crucial for ensuring the satisfactory service performance of components.

TTT and TTP curves are frequently employed to gauge premature precipitation, facilitating the study of alloy quench sensitivity [10,11,12]. According to the TTT and TTP curves, the nose temperature for most intense precipitation of the alloy can be obtained. Xie et al. developed TTT and TTP curves for 7079 aluminum alloy [13]. Their findings indicated a quench sensitivity temperature range for the alloy of 230–370 °C, with a nose temperature at 320 °C. Li et al. examined the impact of alloy composition on the quench sensitivity of 7085 aluminum alloy [14]. Their results demonstrated a significant increase in the nose temperature and critical time of the alloy with higher concentrations of major elements (Zn, Mg, and Cu). Zheng et al. investigated the influence of homogenization time on the quench sensitivity of 7085 aluminum alloy, noting a slight increase in quench sensitivity with prolonged homogenization [15]. This effect may be attributed to the extended homogenization, which facilitates the dissolution of coarser second phase particles. The investigation of quench sensitivity plays a pivotal role in enhancing the quenching process. Nevertheless, to our knowledge, no studies on the quench sensitivity of 7A65 aluminum alloy have been conducted as of yet.

As a new generation of Al-Zn-Mg-Cu aluminum alloy, there is presently no relevant research on the quenching sensitivity of 7A65 aluminum alloy. Therefore, in this study, the TTT and TTP curves were constructed to assess the alloy’s quenching sensitivity through interrupted quenching experiments. The nose temperature and the sensitive temperature range of the alloy were determined. The microstructural evolution of the samples during isothermal treatment was investigated through TEM observations and XRD tests. Subsequently, the mechanism of premature precipitation was analyzed based on the principles of phase transformation kinetics. Ultimately, appropriate quenching process parameters were established. This research offers data and theoretical underpinning for the fabrication of thick plates from 7A65 aluminum alloy and could serve as a guide for selecting quenching parameters in the case of other Al-Zn-Mg-Cu aluminum alloys.

## 2. Material and Experiments

Experimental samples were prepared from a pre-stretched commercial 7A65 aluminum alloy sheet, shaped into dimensions of 15 × 15 × 4 mm. Following a solution treatment at 472 °C for 1 h, the samples were subjected to isothermal treatment in a salt bath furnace and subsequently water-quenched at room temperature. Isothermal treatment was performed across a range of temperatures (240–440 °C) and for varying durations (5–1200 s). The sample transfer time did not exceed 2 s, and the salt bath temperature remained within a ±2 °C range. Following water quenching, the samples underwent conductivity testing using a D60K conductivity tester within 15 min to mitigate the influence of natural aging. Subsequently, the samples underwent a single-stage aging process at 120 °C for 24 h, followed by hardness testing. The hardness of the samples was measured using the HV-1000A microhardness tester with an applied load of 200 g for 15 s, and sample underwent at least five tests to ensure data accuracy.

The microstructure evolution of the samples during isothermal treatment was assessed through X-ray diffraction (XRD) tests and transmission electron microscope (TEM) observation. The XRD tests were conducted using the D8 Advance DAVINC X-ray polycrystalline diffractometer. The TEM samples were initially mechanically polished to a thickness of 75 μm and then punched into 3 mm diameter discs. Subsequently, double-jet polishing was performed using a solution comprising 30% nitric acid and 70% methanol at approximately −25 °C.

## 3. Results

### 3.1. Conductivity Evolution and TTT Curve of 7A65 Aluminum Alloy

**Figure 1:**
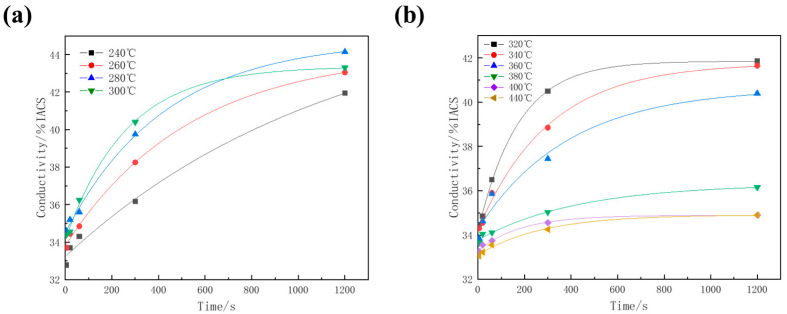
Influence of isothermal treatment parameters on conductivity of quenched samples: (**a**) 240~300 °C; (**b**) 320~440 °C.

As shown in Figure 1, the electroconductivity of the samples after isothermal holding at 240~440 °C for different times was measured. The electrical conductivity experienced rapid initial growth followed by a gradual rise to a stable level. The rate of conductivity growth at different temperatures exhibited some variation. As depicted in Figure 1a, in the lower temperature range, the rate of conductivity growth increased with rising temperature. For instance, when the samples underwent isothermal treatment at 240 °C for 300 s, the conductivity only increased by approximately 10%, whereas at 300 °C for 300 s, the increase was about 14%. Conversely, after isothermal treatment at higher temperatures, the growth rate of the alloy’s conductivity displayed a decreasing trend as the temperature increased with extended holding time (Figure 1b). For example, when the samples underwent isothermal treatment at 320 °C, the conductivity exhibited rapid growth with extended holding time. After holding at 320 °C for 60 s, the conductivity increased by approximately 5.9%, and it increased by nearly 17% as the holding time extended to 300 s. However, after isothermal treatment at 440 °C for 300 s, the conductivity of the alloy increased by only 3.6%. It is believed that the evolution of electrical conductivity can characterize the transformation of solid solution [16]. Generally, the electrical conductivity consistently increases with prolonged isothermal treatment. Consequently, the electrical conductivity of the alloy that was directly quenched after solid solution treatment (*γ_min_* = 32% IACS) corresponded to the undecomposed state of the supersaturated solid solution. Likewise, the electrical conductivity of the sample subjected to isothermal treatment at 320 °C for 48 h *(γ_max_* = 45.8% IACS) corresponded to the complete decomposition of the supersaturated solid solution.

**Figure 2:**
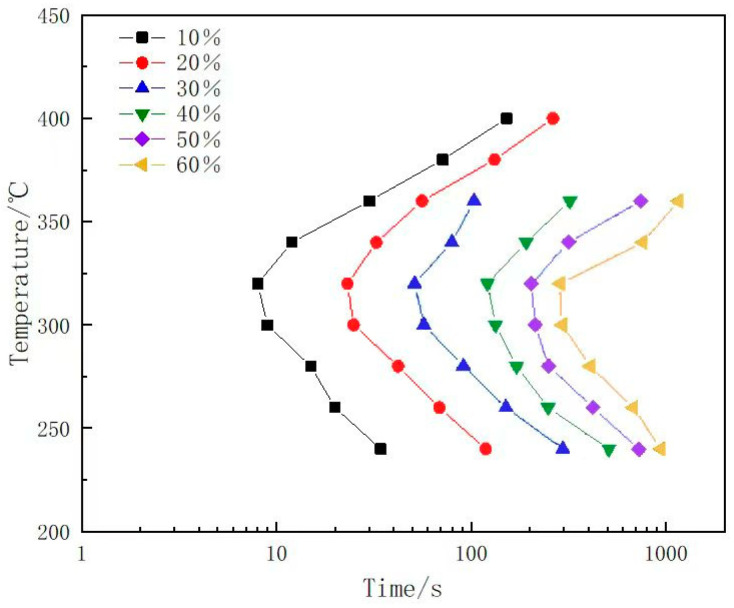
The TTT curves of 7A65 aluminum alloy.

The TTT curve reflected the transformation of the supersaturated solutions, which plays a crucial role in studying the premature precipitation behavior of the alloy during isothermal treatment. The transformation of supersaturated solution is illustrated by Equation (1):

(1) $$f=\frac{\gamma -\gamma_{min}}{\gamma_{max}-\gamma_{min}}$$

where *f* is the transformation fraction, *γ* is the conductivity of the samples, *γ_min_* is the minimum conductivity (32% IACS), and *γ_max_* is the maximum conductivity (45.8% IACS). The holding times corresponding to 10%, 20%, 30%, 40%, 50%, and 60% transformation of supersaturated under different isothermal temperatures can be calculated by interpolation. Subsequently, by connecting the temperature (T)–time (t) points with the same fraction, the TTT curve of the alloy can be constructed, as shown in Figure 2. It can be seen that the TTT curve of the alloy exhibits a “C”-shaped profile, with a nose temperature of approximately 320 °C. The shortest transformation time at the nose temperature leads to an extremely rapid decomposition of the supersaturated solution, resulting in high quench sensitivity. In contrast, the longer transformation times in the high-temperature and low-temperature regions lead to a relatively slower decomposition of the supersaturated solution and lower quench sensitivity.

### 3.2. Hardness Evolution and the TTP Curve of 7A65 Aluminum Alloy

**Figure 3:**
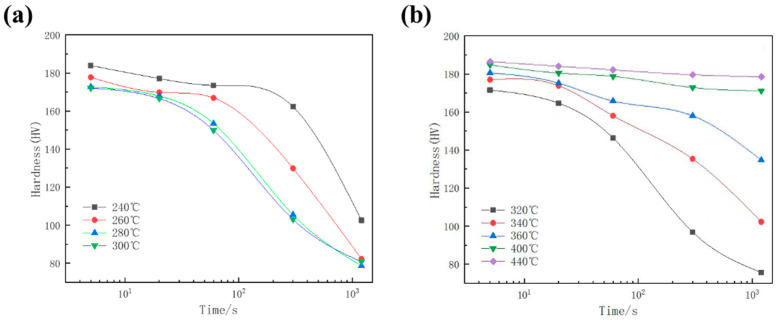
The influence of isothermal treatment on the hardness of the as-aged samples: (**a**) 240~300 °C; (**b**) 320~440 °C.

The evolution of hardness in samples subjected to various isothermal treatments is shown in Figure 3. Evidently, hardness decreased as the isothermal holding time was extended. Although all temperatures exhibited a decreasing trend, the rate of hardness decrease varied among samples at different temperatures. Within the range of 240–320 °C, the rate of hardness decrease in the as-aged sample increased with the extension of isothermal holding time and temperature. Following a 300 s hold at 240 °C, the alloy’s hardness decreased by only approximately 11.7%. In contrast, the hardness of the alloy decreased significantly with prolonged time at 320 °C. After a 60 s hold at 320 °C, the hardness of the alloy dropped by nearly 15.0%. With a 300 s hold, the hardness decreased by about 43.5%. However, as the temperature exceeded 320 °C, the hardness decrease rate reduced with rising temperature. At 420 °C, the hardness of the alloy decreased at a slower rate with the extension of holding time, and samples isothermally held for 1200 s only experienced a 4% decrease in hardness.

**Figure 4:**
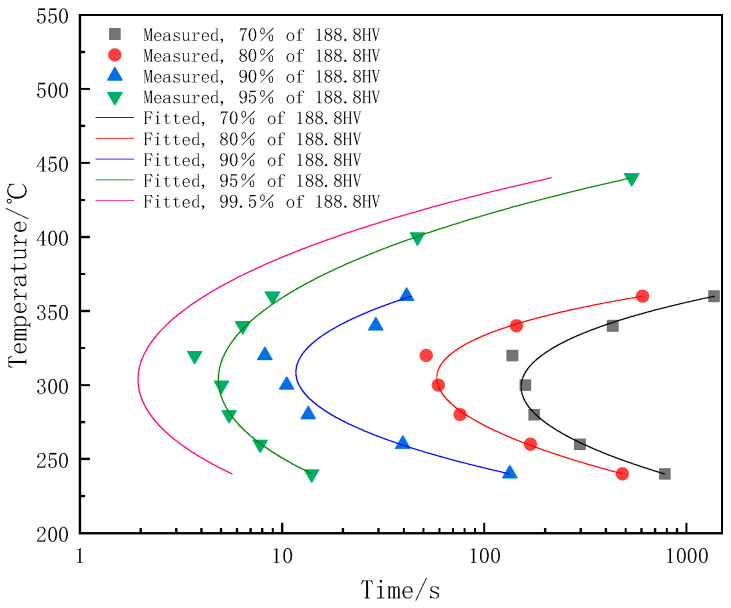
The TTP curve of 7A65 aluminum alloy.

The TTP curve is typically employed to depict the correlation between material properties, isothermal treatment temperature, and isothermal treatment time, and it can be fitted according to Equation (2) [17,18]:

(2) $$C\left(T\right)=-k_{1}k_{2}\exp\left(\frac{k_{3}k_{4}^{2}}{RT{\left(k_{4}-T\right)}^{2}}\right)\exp\left(\frac{k_{5}}{RT}\right)$$

where *C* denotes the critical time required for the transformation of the supersaturated solution; *k*_1_ represents the natural logarithm of the untransformed fractions; *k*_2_ is the constant associated with the reciprocal of the number of nucleation sites; *k*_3_ stands for the constant related to the energy needed for nucleus formation; *k*_4_ is the constant linked to the solvus temperature; *k*_5_ signifies the constant associated with the activation energy for diffusion; *R* denotes the molar gas constant (8.314 J/mol·K); and *T* represents the absolute temperature of the alloy in K. As shown in Figure 4, the TTP curve of the alloy was fitted, and the fitted parameters were shown in Table 1. The sample aged directly after quenching was considered without premature precipitation and the hardness (188.8 HV) was used as a reference. It can be seen that the fitted curves precisely anticipate alterations in alloy properties. The nose temperature of the 7A65 aluminum alloy is approximately 320 °C, consistent with the TTT curve. Furthermore, considering the attainment of 0.5% transformation within 10 s, the sensitive temperature range of the alloy spans from 240 to 360 °C. The mechanical properties of the alloy degrade rapidly when held within the sensitive temperature ranges.

### 3.3. Microstructure Evolution during Isothermal Treatment

**Figure 5:**
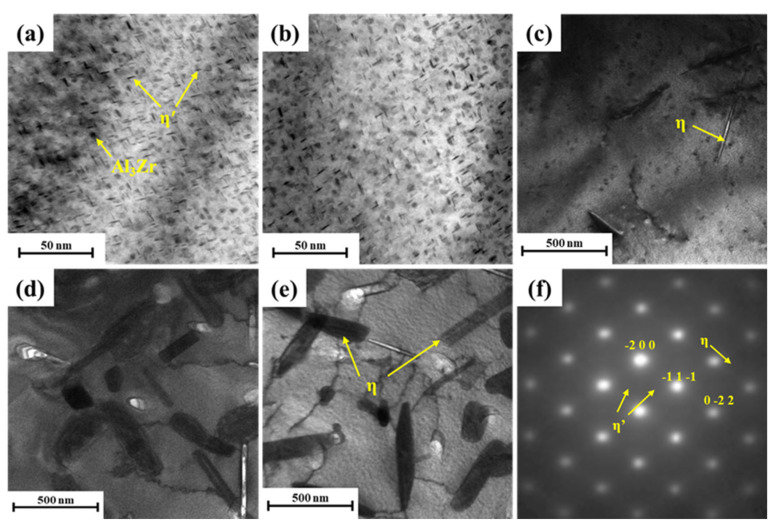
TEM images in matrix of the as-aged samples isothermal-treated at 320 °C with different holding times: (**a**) 5 s, (**b**) 20 s, (**c**) 60 s, (**d**) 300 s, (**e**) 1200 s, (**f**) SAED image.

To investigate the microstructural evolution of the samples during isothermal treatment, TEM observations were carried out along the <110>_Al_ direction. The microstructural evolution in the matrix of the as-aged samples subjected to isothermal treatment at the nose temperature is shown in Figure 5. It is widely accepted that precipitation in 7XXX series aluminum alloys occurs in the following sequence: supersaturated solid solution status (SSSS) → clusters/Guinier–Preston zone (GP zone) → metastable phase η’ → equilibrium phase η (MgZn_2_) [19,20,21]. Numerous nanoscale η′ phases were observed within the grains of samples with short isothermal times, along with spherical Al_3_Zr particles (Figure 5a,b). These Al_3_Zr particles serve as sites for heterogeneous nucleation, promoting the precipitation and growth of phases. When the isothermal annealing time was extended to 60 s, rod-shaped η phases (MgZn_2_) appeared within the grains, which precipitated during the isothermal process before aging (Figure 5c). With the holding time further extended to 300 s, the number and size of rod-shaped η phases within the grains increased (Figure 5d). Extending the isothermal time to 1200 s resulted in the continued growth of the η phase, reaching a size of approximately 480–650 nm (Figure 5e). A notable decrease in the nanoscale phases surrounding the η phases was observed. This is attributed to premature precipitation reducing the supersaturation of the alloy and inhibiting subsequent precipitation during aging. In addition, the identification of phase evolution during isothermal treatment is based on selected area electron diffraction (SAED) (Figure 5f).

**Figure 6:**
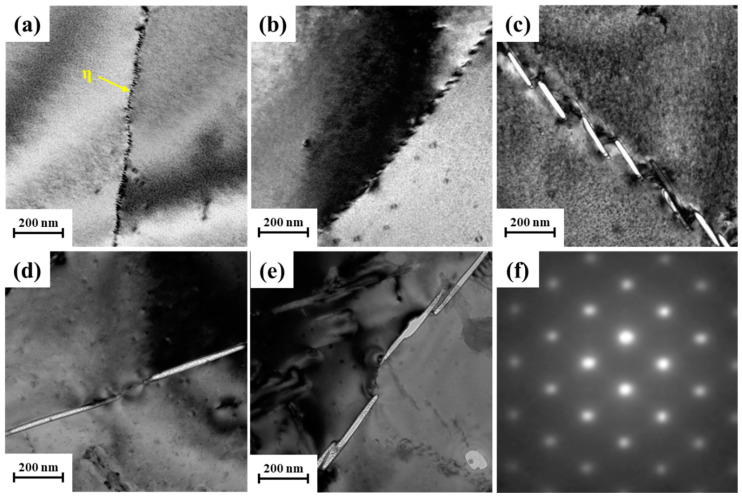
TEM images around the grain boundary of the as-aged samples isothermally treated at 320 °C with different holding times: (**a**) 5 s, (**b**) 20 s, (**c**) 60 s, (**d**) 300 s, (**e**) 1200 s, (**f**) SAED image.

The microstructure at grain boundaries is shown in Figure 6 After isothermal treatment for 5 s, the η phase at grain boundaries is small and exhibits a discontinuous distribution (Figure 6a). These equilibrium η phases hinder the precipitation of the η′ phase around the grain boundaries, resulting in the formation of a precipitation-free zone (PFZ). Extending the holding time to 20 s leads to the gradual growth of phases at grain boundaries while maintaining their discontinuous nature, and the spacing between phases widens (Figure 6b). Upon reaching an isothermal time of 60 s, the η phases at grain boundaries continue to thicken and lengthen, and the PFZ significantly widens (Figure 6c). With an extension of the holding time to 300 s, the length of the phases reached approximately 400 nm, and there was no significant change in morphology with the increasing holding time (Figure 6d,e). These larger and coarser rod-shaped precipitates accumulate at the grain boundaries, adversely affecting the mechanical properties of the alloy, notably resulting in a significant reduction in corrosion resistance [22,23].

## 4. Discussion

**Figure 7:**
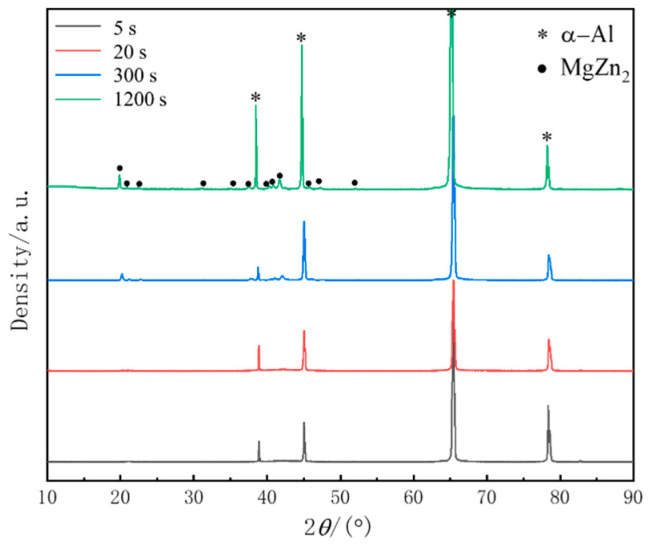
XRD patterns of the quenched samples after isothermal treatment at 320 °C for different times.

According to the analysis in the previous sections, the premature precipitation during isothermal processes can seriously affect the mechanical properties of the components. A large number of equilibrium phases are precipitated during isothermal treatment with increasing the holding time, coarse especially at the nose temperature. The XRD results of the quenched samples after isothermal treatment at 320 °C for different times are shown in Figure 7. It can be seen that the peak intensity of η phase (MgZn_2_) increases gradually with isothermal time. The MgZn_2_ peaks were not observed of the sample before isothermal treatment for 20 s. When the holding time was extended to 300 s, the intensity of MgZn_2_ peaks was significantly enhanced, which indicates that a large number of equilibrium phases were precipitated in the matrix. When the holding time was extended to 1200 s, there was no significant increase in peak intensity, which indicates that MgZn_2_ was fully precipitated, which is consistent with the TEM results (Figure 6).

The quenching sensitivity of the alloy is influenced by crucial factors such as composition and nucleation location. The nucleation rate I of the alloy can be expressed by Equation (3) [24]:

(3) $$I=A\exp\left(-\frac{\Delta G^{*}+Q}{kT}\right)$$

where *A* represents a constant; ΔG* is the activation energy for nucleation; *Q* represents the activation energy for solute diffusion; *k* is the Boltzmann constant (1.38 × 10^−23^ J/K); and *T* is the absolute temperature of the alloy. In the low-temperature range, the solid solution has high supersaturation, which leads to a higher precipitation power and a large value of ΔG*. The diffusion of solute atoms is impeded at lower temperatures, resulting in a reduction in *Q* and a consequential significant decrease in the nucleation rate of the alloy. Conversely, higher temperatures lead to an increase in the diffusion rate of solid solute atoms, but the nucleation activation energy is reduced due to the lower supersaturation, resulting in a decrease in the nucleation rate of the alloy. However, the medium temperature range exhibits a relatively high supersaturation of the alloy and diffusion rate of solute atoms, thereby promoting rapid nucleation, growth, and transformation of phases in the alloy.

**Figure 8:**
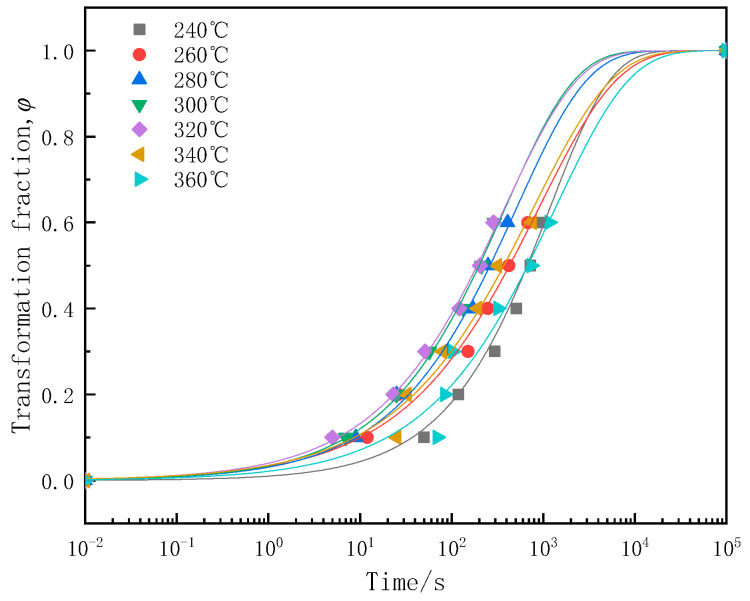
The time-dependent transformation fraction curve of 7A65 aluminum alloy.

**Figure 9:**
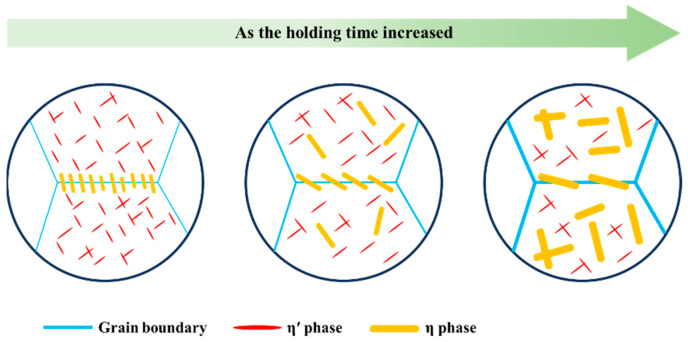
Precipitation scenario for the as-aged samples after isothermal treatment at 320 °C for different times.

The Avrami phase transition kinetic equation was fitted according to the change in conductivity of the sample, as shown in Equation (4) [25,26,27]:

(4) $$\varphi =1-\exp\left(-kt^{n}\right)$$

where *φ* represents the phase transformation fraction, *k* is a constant related to nucleation and growth rates, *t* signifies the isothermal time, and *n* is contingent on the precipitation mechanism. The isothermal transformation curves for 7A65 aluminum alloy are presented in Figure 8, and the corresponding equation parameters are detailed in Table 2. It is evident that, as the temperature rises, the rate of supersaturated solution transformation initially increases and then decreases, with the *k* value peaking at 320 °C, which is consistent with the experimental results. Furthermore, the emergence of plate-like or needle-like phases within the grain is governed by the parameter *n*, wherein a value proximal to 0.5 signifies the prevalence of the former, while a value approaching 1 signifies the predominance of the latter [28]. The range of fitted *n* values is 0.5–0.7, which coincides with the TEM observations. The rapid transformation of the supersaturated solution at the nose temperature results in a substantial depletion of solute atoms and the swift precipitation of coarse equilibrium phases within the matrix, significantly impacting the mechanical properties of the component. Based on the experimental results, the scenario of precipitation behavior of the as-aged 7A65 aluminum samples isothermally treated at the nose temperature is shown in Figure 9. During short isothermal treatments, only a limited number of fine equilibrium η phases form at the grain boundaries, typically oriented perpendicularly to the grain boundaries. Due to the short holding time, supersaturation degree is still high and a large number of nanoscale η′ phases can be precipitated in the matrix during the subsequent aging process. As the isothermal time increases, the η phases at the grain boundaries coarsen and align parallel to the grain boundary. Additionally, coarse equilibrium phases develop within the grain boundaries, signifying significant premature precipitation. The solute atoms are notably depleted during isothermal treatment, particularly in the vicinity of the η phases, resulting in diminished quantities of nanoscale η′ phases and a subsequent reduction in the mechanical properties of the as-aged samples.

## 5. Conclusions

In this study, the quench sensitivity of 7A65 aluminum alloy was investigated. The TTT and TTP curves of the alloy were established, and the nose temperature was determined. Further, the premature precipitation mechanism was discussed in detail based on the TEM observation results. The main conclusions are summarized as follows:

1. Severe premature precipitation occurred during isothermal treatment, and the nose temperature of the 7A65 aluminum alloy is 320 °C and the sensitive temperature range is from 240 °C to 360 °C. After isothermal treatment at 320 °C for only 60 s, the hardness of the sample decreased by nearly 15%.
2. The η phases precipitated rapidly and grew within both the matrix and at the grain boundaries during isothermal treatment at the nose temperature. This severe premature precipitation depletes solute atoms, resulting in reduced quantities of nanoscale η′ phases and a broader PFZ.
3. To achieve a balance between the mechanical properties and residual stresses of 7A65 aluminum alloy, it is necessary to increase the quenching cooling rate in the temperature range from 240 to 360 °C and appropriately reduce the cooling rate from the solid solution temperature to 360 °C.

## Figures and Tables

**Table 1:** The TTP curve equation parameters of 7A65 aluminum alloy.

Coefficient	k_2_ (s)	k_3_ (J/mol)	k_4_ (K)	k_5_ (J/mol)
Value	1.4 × 10^−11^	21,838	822	9381

**Table 2:** The fitted parameters of the Avrami equation.

Temperature/°C	k	*n*
240	0.01000	0.65146
260	0.03270	0.50379
280	0.03138	0.55567
300	0.03478	0.56484
320	0.04096	0.53904
340	0.03572	0.50111
360	0.02155	0.53257

## Data Availability

Data are contained within the article.

## References

[1] Beura V.K., Sharma A., Karanth Y., Sharma S., Solanki K. (2023). Corrosion behavior of 7050 and 7075 aluminum alloys processed by reactive additive manufacturing. Electrochim. Acta.

[2] Peterson L.A., Horstemeyer M.F., Lacy T.E., Moser R. (2020). Experimental characterization and constitutive modeling of an aluminum 7085-T711 alloy under large deformations at varying strain rates, stress states, and temperatures. Mech. Mater..

[3] Carvalho A.L.M., Renaudin L.B., Zara A.J., Martins J. (2022). Microstructure analysis of 7050 aluminum alloy processed by multistage aging treatments. J. Alloys Compd..

[4] Korbel A., Bochniak W., Borowski J., Błaż L., Ostachowski P., Łagoda M. (2015). Anomalies in precipitation hardening process of 7075 aluminum alloy extruded by KOBO method. J. Mater. Process. Technol..

[5] Engler O. (2022). Effect of precipitation state on plastic anisotropy in sheets of the age-hardenable aluminium alloys AA 6016 and AA 7021. Mater. Sci. Eng. A.

[6] Ahmad A.S., Yunxin W., Hai G., Liu L. (2019). Determination of the Effect of Cold Working Compression on Residual Stress Reduction in Quenched Aluminium Alloy 2219 Block. Stroj. Vestn. J. Mech. Eng..

[7] Lim H., Ko D., Ko D., Kim B.M. (2014). Reduction of Residual Stress and Improvement of Dimensional Accuracy by Uphill Quenching for Al6061 Tube. Metall. Mater. Trans. B Process Metall. Mater. Process. Sci..

[8] Olson M.D., Robinson J.S., Wimpory R.C., Hill M.R. (2016). Characterisation of residual stresses in heat treated, high strength aluminium alloy extrusions. Mater. Sci. Technol..

[9] Murayama M., Hono K. (1999). Pre-precipitate clusters and precipitation processes in Al-Mg-Si alloys. Acta Mater..

[10] Archambault P., Godard D. (2000). High temperature precipitation kinetics and ttt curve of a 7xxx alloy by in-situ electrical resistivity measurements and differential calorimetry. Scr. Mater..

[11] Evancho J.W., Staley J.T. (1974). Kinetics of precipitation in aluminum alloys during continuous cooling. Metall. Trans..

[12] Davydov V.G., Ber L.B., Kaputkin E.Y., Komov V.I., Ukolova O.G., Lukina E.A. (2000). TTP and TTT diagrams for quench sensitivity and ageing of 1424 alloy. Mater. Sci. Eng. A.

[13] Xie P., Chen K., Chen S., Ye S., Jiao H., Huang L. (2020). Study on quenching sensitivity of 7097 aluminum alloy. Mater. Res. Express.

[14] Li C., Chen D. (2019). Investigation on the Quench Sensitivity of 7085 Aluminum Alloy with Different Contents of Main Alloying Elements. Metals.

[15] Zheng Y., Li C., Liu S., Deng Y.L., Zhang X.M. (2014). Effect of homogenization time on quench sensitivity of 7085 aluminum alloy. Trans. Nonferrous Met. Soc. China.

[16] Liu S., Wang X., Pan Q., Li M., Ye J., Li K., Peng Z., Sun Y. (2020). Investigation of microstructure evolution and quench sensitivity of Al–Mg–Si–Mn–Cr alloy during isothermal treatment. J. Alloys Compd..

[17] Tiryakioğlu M., Shuey R.T. (2010). Quench sensitivity of 2219-T87 aluminum alloy plate. Mater. Sci. Eng. A.

[18] Milkereit B., Starink M.J. (2015). Quench sensitivity of Al-Mg-Si alloys: A model for linear cooling and strengthening. Mater. Des..

[19] Lervik A., Marioara C.D., Kadanik M., Walmsley J.C., Milkereit B., Holmestad R. (2020). Precipitation in an extruded AA7003 aluminium alloy: Observations of 6xxx-type hardening phases. Mater. Des..

[20] Zhao H., Gault B., Ponge D., Raabe D. (2020). Reversion and re-aging of a peak aged Al-Zn-Mg-Cu alloy. Scr. Mater..

[21] Bendo A., Matsuda K., Nishimura K., Nunomura N., Tsuchiya T., Lee S., Marioara C., Tsuruc T., Yamaguchi M., Shimizud K. (2020). The possible transition mechanism for the meta-stable phase in the 7xxx aluminium. Mater. Sci. Technol..

[22] Knight S.P., Birbilis N., Muddle B.C., Trueman A.R., Lynch S.P. (2010). Correlations between intergranular stress corrosion cracking, grain-boundary microchemistry, and grain-boundary electrochemistry for Al-Zn-Mg-Cu alloys. Corros. Sci..

[23] Ralston K.D., Birbilis N., Weyland M., Hutchinson C.R. (2010). The effect of precipitate size on the yield strength-pitting corrosion correlation in Al-Cu-Mg alloys. Acta Mater..

[24] Wang H., YI Y., Huang S. (2017). Investigation of quench sensitivity of high strength 2219 aluminum alloy by TTP and TTT diagrams. J. Alloys Compd..

[25] Avrami M. (1939). Kinetics of Phase Change. I General Theory. J. Chem. Phys..

[26] Avrami M. (1940). Kinetics of Phase Change. II Transformation-Time Relations for Random Distribution of Nuclei. J. Chem. Phys..

[27] Avrami M. (1941). Granulation, Phase Change, and Microstructure Kinetics of Phase Change. III. J. Chem. Phys..

[28] Christian J.W. (2002). The Theory of Transformations in Metals and Alloys, PARTI.

